# Functional Connectivity Abnormalities of Brain Regions With Structural Deficits in Primary Insomnia Patients

**DOI:** 10.3389/fnins.2020.00566

**Published:** 2020-06-26

**Authors:** Dongdong Xie, He Qin, Fang Dong, XianFu Wang, Chang Liu, Ting Xue, Yifu Hao, Bo Liu, Kai Yuan, Dahua Yu

**Affiliations:** ^1^Inner Mongolia Key Laboratory of Pattern Recognition and Intelligent Image Processing, School of Information Engineering, Inner Mongolia University of Science and Technology, Baotou, China; ^2^Department of Neurology, Affiliated Hospital of Inner Mongolia University for the Nationalities, Tongliao, China; ^3^School of Life Science and Medicine Bioinformatics, Dalian University of Technology, Dalian, China; ^4^Department of Neurology, The First Affiliated Hospital of Baotou Medical College, Inner Mongolia University of Science and Technology, Baotou, China; ^5^Life Sciences Research Center, School of Life Sciences and Technology, Xidian University, Xi’an, China

**Keywords:** primary insomnia, voxel-based morphometry (VBM), resting state functional connectivity, middle frontal gyrus, inferior frontal gyrus

## Abstract

**Objectives:**

The present study examined the abnormal resting state functional connections (RSFCs) in structural deficit brain regions of primary insomnia (PI) patients.

**Methods:**

Thirty-three PI patients and 38 well-matched healthy controls participated in our study. We used voxel-based morphometry and RSFC to study functional connectivity abnormalities of brain regions with structural deficits in PI patients.

**Results:**

PI patients showed decreased gray matter (GM) volume in the left dorsolateral prefrontal cortex, left orbitofrontal cortex (OFC), bilateral middle frontal gyrus (MFC), right inferior frontal gyrus (IFG), and left inferior temporal gyrus. Gray matter volume in the right MFC negatively correlated with Self-Rating Scale of Sleep (SRSS) scores, and GM volume in the right IFG negatively correlated with SRSS and Insomnia Severity Index (ISI) scores. Therefore, the right MFC and right IFG were selected as regions of interest for RSFC analysis. PI patients had weakened RSFC between the right inferior parietal gyrus (IPC) and the right MFC compared to the healthy controls and between the left OFC and right IFG. The RSFC between the right MFC and right IPC negatively correlated with SRSS scores. The RSFC between the right IFG and left OFC negatively correlated with SRSS, ISI, SAS, and SDS scores.

**Conclusions:**

The present study found structural changes in the right MFC and right IFG accompanied by RSFC changes. This finding may provide novel insights into the neural mechanisms of PI *via* combining structural and functional modality information.

## Introduction

As one of the most common health problems in people and in clinical practice (Lichstein et al., 2003), primary insomnia (PI) generally manifests as difficulties in the onset and maintenance of sleep (Morin et al., 2011). Primary insomnia is present in 6–10% of the total population, especially women and older adults (Xu et al., 2011; Bélanger et al., 2012; Buysse, 2013), which may influence their quality of life and produce physical impairment, mental disorders, and increased medical care costs (Buysse, 2013). Over the past few decades, many neuroimaging studies investigated the neural mechanism of PI and found extensive structural and functional changes in several brain regions. Unfortunately, the neurobiological mechanism of PI is not clear.

Brain imaging technology is a useful tool for studying the neural mechanism of PI. Many structural neuroimaging studies used voxel-based morphometry (VBM) analysis to reveal PI-related brain tissue injury (Yeon et al., 2013), and multiple brain regions showed abnormal gray matter (GM) volume. Primary insomnia patients revealed decreased GM volume in the hippocampus, precuneus, middle temporal gyrus, left orbitofrontal cortex (OFC), the bilateral dorsolateral prefrontal cortex (DLPFC), left middle frontal gyrus (MFC), and the bilateral inferior frontal gyrus (IFG; Dieter et al., 2007; Altena et al., 2010; Stoffers et al., 2012; Joo et al., 2013; Li et al., 2018) compared to healthy controls. Functional neuroimaging methods were also used to study brain functional abnormality in PI patients. Resting state functional connectivity (RSFC) is primarily used to examine spontaneous neuronal activity in the human brain, which may provide new insights into the neurological mechanism of abnormal behavior in PI patients (Dijk et al., 2010; Friston, 2011). Seed-based connectivity analysis revealed decreased RSFC between the parietal lobe and the frontal lobe (Li et al., 2014) and between the left OFC and left caudate (Diederick et al., 2014). Our previous study found reduced RSFC between the thalamus and hippocampus, anterior cingulate cortex (ACC), OFC, caudate, and putamen (Min et al., 2018). Most previous studies focused on the difference in RSFC between PI patients and healthy subjects, but few studies investigated the RSFC in brain structural defect regions of PI patients in the whole brain.

The structural changes may be accompanied by impairment of brain function. Multimodal technical methods that combine structure and function may provide more useful information for clinical diagnosis. The combination of VBM and FC was used to study schizophrenia (Zhang et al., 2015), Parkinson’s disease (Canu et al., 2015), and temporal lobe epilepsy (Doucet et al., 2016). To our knowledge, no previous study has investigated the neurobiological mechanisms of PI using a combination of VBM and FC analysis. The present study detected common and distinct brain structural and functional alterations between PI patients and healthy controls based on brain structural and functional images. The current study enrolled relatively homogeneous subjects to investigate structural abnormalities between PI patients and a control group. Changes in brain RSFC with structural deficits were assessed. We hypothesized that brain regions with structural deficits in PI patients would show abnormal RSFC, and these changes would be associated with sleep behaviors. The combination of structural and functional methods may provide new perspectives on the neural mechanism of PI.

## Materials and Methods

The Medical Ethics Committee of the First Affiliated Hospital of Baotou Medical College, Inner Mongolia University of Science and Technology approved all procedures. Before the experimental study, a researcher informed the participants of the possible risks and discomfort of participating in the study. All of the participants volunteered to participate in the study and provided written informed consent.

### Participants

Subjects were recruited from the First Affiliated Hospital of Baotou Medical College, Inner Mongolia University of Science and Technology, Baotou, China. Thirty-three adults with PI (10 males, 23 females; mean ± standard deviation age = 42.27 ± 9.27 years) and 38 age-, gender-, education-matched healthy control subjects (15 males, 23 females; mean ± standard deviation age = 43.72 ± 10.10 years) participated in the present study. The following inclusion criteria for PI patients were used: (1) age 18–68 years; (2) right-handed, Han nationality; (3) meeting the criteria for sleep disturbance in the DSM-V and lasting for more than 3 months; (4) reporting difficulty in initiating and maintaining sleep or early awakening for at least 1 month; and (5) PSQI total score ≧ 8. The following exclusion criteria were used: (1) intellectual disability, mental disorders (such as epilepsy) or chronic pain diseases; (2) patients with serious primary diseases, such as cancer, diseases of the heart, liver, lung, kidney, hematopoietic system, and other diseases; (3) pregnant or lactating women, allergic constitution and allergic to a variety of drugs; (4) MRI examination showed organic occupying, bright signal or other structural abnormalities in the brain; (5) medication or substance abuse, such as alcohol, nicotine, or other drugs; and (6) MRI contraindications (cardiac pacemaker, insulin pump, artificial heart valve, and others with metal in the body, or claustrophobia). The following inclusion criteria were used for healthy controls: (1) good sleep quality, no difficulty in starting or maintaining sleep; and (2) PSQI total score <5. The exclusion criteria were the same as the PI patients. All subjects completed a number of self-questionnaires, such as the Pittsburgh Sleep Quality Index (PSQI), Insomnia Severity Index (ISI), Self-Rating Scale of Sleep (SRSS), Self-Rating Anxiety (SAS), and Self-Rating Depression Scale (SDS). The study protocol was performed in compliance with the Declaration of Helsinki and approved by the research Ethical Committee of First Affiliated Hospital of Baotou Medical College, Inner Mongolia University of Science and Technology.

### Data Acquisition

Magnetic resonance imaging (MRI) was performed using a 3T Philips scanner (Achieva; Philips Medical Systems, Best, Netherlands) at the First Affiliated Hospital of Baotou Medical College, Inner Mongolia University of Science and Technology, Baotou, China. The head of the subject was fixed in a foam pad and kept in a comfortable position before the scan. Subjects wore earplugs to reduce the loud noise from the machine. A high-resolution T1 structural image was obtained using a magnetization-prepared rapid acquisition gradient echo (MPRAGE) pulse sequence and the following scanning parameters: repetition time/echo time (TR/TE) = 8.5/3.4 ms; matrix = 240 × 240; slices = 140; and field of view (FOV) = 240 mm × 240 mm; and voxel size = 1 mm × 1 mm × 1 mm. Resting state functional images were scanned using an echo-planar imaging (EPI) sequence (slices = 32; slice thickness = 5 mm; TR/TE = 2000/30 ms; flip angle (FA) = 90°; FOV = 240 mm × 240 mm; data matrix = 64 × 64; total volumes = 185). Subjects remained awake with their eyes closed during the entire scan and were told not to think about anything. At the end of scanning, the participants were asked if they were awake during the functional data collection. Two radiologists examined the images of all participants to exclude any aspect of clinical pathological changes in any subject.

### Data Analysis

#### VBM Analysis

All T1 data preprocessing were performed using the Statistical Parametric Mapping 8 software (SPM8; Wellcome Department of Cognitive Neurology) and VBM8 toolbox (University of Jena, Department of Psychiatry) run in Matlab2011a. First, the original T1 data were registered to the Montreal Neurological Institute (MNI) template to match the brain structure images of different subjects in the same space. Subsequently, the structure images were segmented into GM, white matter (WM), and cerebrospinal fluid (CSF) using an integrated one-pass procedure (the unified segmentation). The segmented GM images were modulated to correct the volume change after standardization. Finally, GM images were smoothed with an 8-mm full-width at half-maximum Gaussian kernel to reduce noise and to improve the signal-to-noise ratio. To compare changes in GM volume between PI patients and healthy controls, a two-sample t-*t*est was performed in SPM8 [familywise error (FWE) correction, *p* < 0.05].

#### RSFC Analysis

Resting state data preprocessing was performed using Analysis of Functional NeuroImages (AFNI)^1^ and FMRIB Software Library (FSL)^2^ as described in our previous studies (Bi et al., 2016; Yuan et al., 2016a, b; Min et al., 2018). Functional data preprocessing was divided into core image processing and denoising. In general, the preprocessing procedure of core images included the following steps: (Lichstein et al., 2003) removal of the first 5 volumes of the functional image; (Morin et al., 2011) slice timing correction; (Bélanger et al., 2012) head motion correction (3° rotations and 3-mm displacements); (Xu et al., 2011) obliquity transformation to the structural image; (Buysse, 2013) affine co-registration to the skull-stripped structural image; (Yeon et al., 2013) spatial normalization to the MNI152 template; (Altena et al., 2010) spatial smoothing (4 mm full-width at half-maximum Gaussian kernel); and (Li et al., 2018) intensity normalization to a whole-brain median of 1000. Previous studies found that nuisance regression and bandpass filtering alone were generally inadequate to control head motion-induced noise (Power et al., 2012; Patel et al., 2014). Therefore, wavelet denoising is used in the study of RSFC (Patel et al., 2014). The denoising steps included: time series despiking (wavelet domain); nuisance signal regression, including the six motion parameters, their first-order temporal derivatives, WM and CSF signal (14-parameter regression); and a temporal Fourier filter (0.009–0.10HZ).

Brain regions that showed altered GM volume between PI patients and healthy controls were defined as the regions of interests (ROIs). Using these ROIs as seed points, the average time series was extracted as the reference time series, and a figure relevant to functional connectivity was obtained between the average fMRI time series of each ROI and all voxels in the brain using the Resting State fMRI Data Analysis Toolkit (REST1.8)^3^ software (Xiao-Wei et al., 2011). The R value maps were transformed into approximate Gaussian distributions using a Fisher’s *Z* transformation. Finally, two-sample *t*-test for *Z* values was performed to observe these brain regions related to the seed points in SPM8 (*p* < 0.05, FWE corrected).

### Correlation Analysis

Pearson correlation analysis was used to examine the relationship between GM volume and clinical variables (SRSS, PSQI, ISI, SAS, and SDS). Brain regions with abnormal GM volumes were deemed ROIs. The average GM volume of these ROIs were extracted using REST1.8. The correlation analysis compared the mean GM volume and the scale scores using IBM SPSS statistics (version 20.0, SPSS Inc, Chicago, IL, United States). Similarly, REST1.8 software was used to extract the functional connectivity strength and the *Z* value of each ROI from the brain regions with abnormal functional connectivity in the two groups. Pearson analysis was used to investigate the relationships between the clinical measures and *Z* values in PI patients. *P* < 0.05 was considered statistically significant.

### Statistical Analyses

Non-imaging data statistical analyses were performed using SPSS version 20.0. Chi-squared test was used to compare the gender difference in the two groups. Two-tailed two-sample *t*-tests were used for SAS, SDS SRSS, PSQI, and age. The significance level was set at *p* < 0.05.

## Results

### Demographic and Clinical Characteristics

The detailed demographic characteristics in the current study are given in Table 1. There were no significant differences in gender or age (*p* > 0.05) between the PI and control groups. However, the PI patients showed higher SRSS, PSQI, SDS, and SAS scores than controls, as expected (Table 1).

**TABLE 1:** Demographic characteristics of primary insomnia (PI) patients and healthy controls (HC) in the present study.

	PI (*N* = 33)	HC (*N* = 38)	*P* value
Male/female	10/23	15/23	0.651
Age (years)	42.27 ± 9.27	43.72 ± 10.10	0.768
SRSS	34.94 ± 6.92	16.33 ± 2.25	< 0.001
PSQI	13.54 ± 3.54	3.86 ± 2.21	< 0.001
SAS	53.75 ± 10.09	24.31 ± 3.49	< 0.001
SDS	47.04 ± 9.60	16.00 ± 9.77	< 0.001
ISI	17.63 ± 6.30	−	−

*Chi-squared test was used for gender comparisons; two-tailed two-sample *t*-tests were used for SRSS, PSQI, SAS, SDS and age comparisons. The significance level was set as *p* < 0.05. Values are expressed as the means ± standard deviations. SRSS, Self-Rating Scale of Sleep; PSQI, Pittsburgh Sleep Quality Index; SAS, Self-Rating Anxiety; SDS, Self-Rating Depression Scale; ISI: Insomnia Severity Index.*

### VBM Results

**FIGURE 1:**
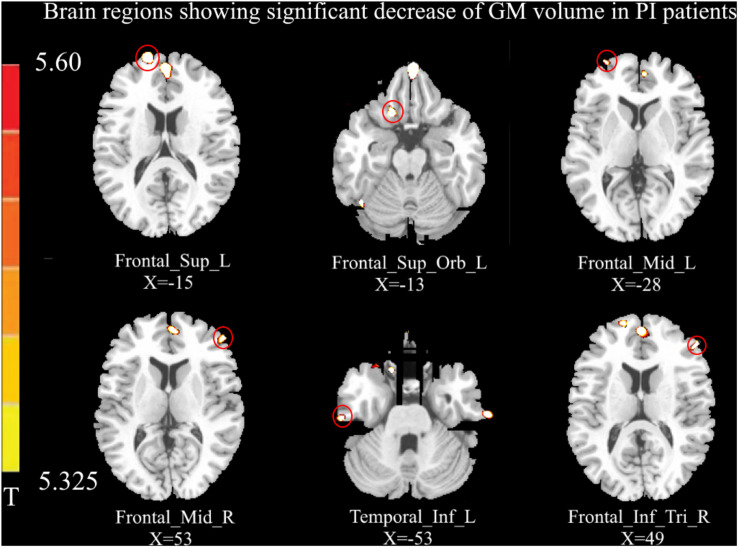
Compared with healthy controls (*p* < 0.05, FEW corrected), the volume of gray matter (GM) in the left dorsolateral prefrontal cortex (DLPFC), left orbital prefrontal cortex (OFC), bilateral middle frontal gyrus (MFC), left inferior temporal gyrus, and right inferior frontal gyrus (IFG) was decreased in primary insomnia (PI) patients. No increased GM volume was observed in the brain regions of PI patients in this study.

**FIGURE 2:**
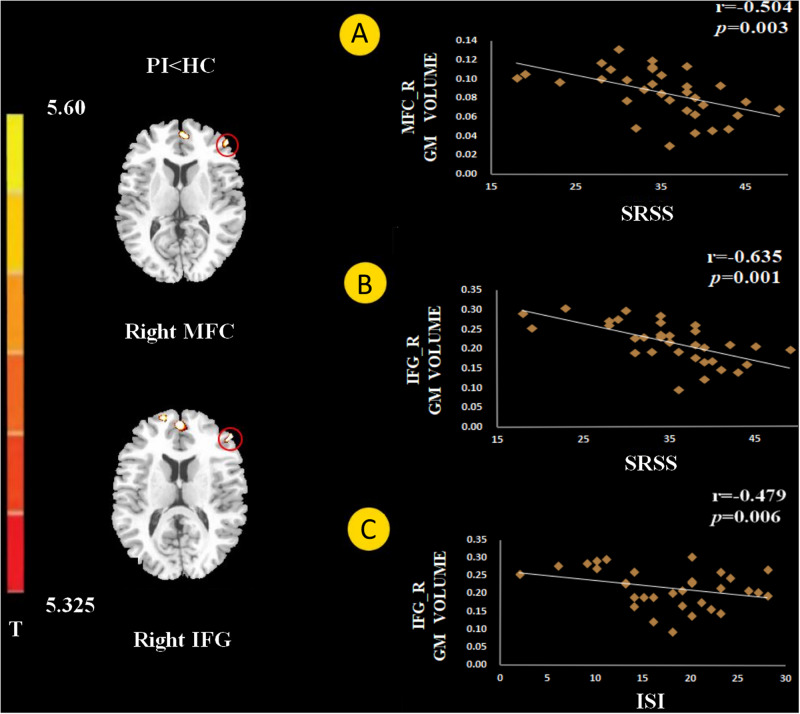
The gray matter (GM) volume in the right middle frontal gyrus (MFC) negatively correlated with Self-Rating Scale of Sleep (SRSS) score **(A)**. The GM volume of the right inferior frontal gyrus (IFG) negatively correlated with SRSS score **(B)**, and Insomnia Severity Index (ISI) score **(C)**.

Relative to healthy controls, the volume of GM in several brain regions was decreased in PI patients, i.e., the left DLPFC, left OFC, bilateral MFC, right IFG, and left inferior temporal gyrus (*p* < 0.05, FWE corrected; Figure 1). However, there was no increase in GM volume in the brain region of PI patients in this study. The correlation analysis of GM volume and behavioral data are shown in Figure 2.

### Functional Connectivity Results

**FIGURE 3:**
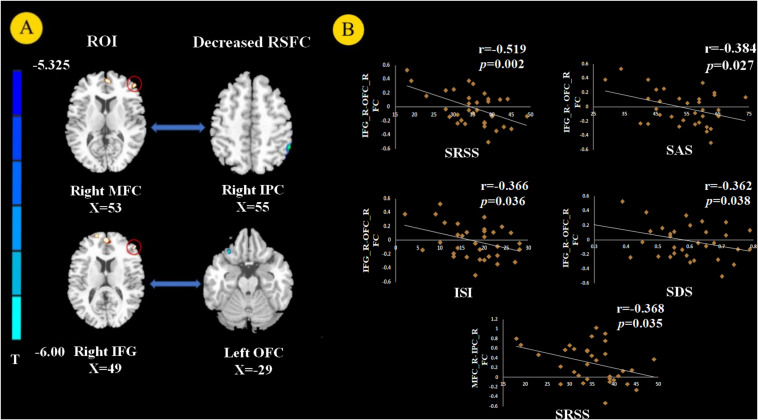
Resting state functional connectivity (RSFC) analysis (*p* < 0.05, FEW corrected). We chose the right MFC and right IFG as the regions of interest (ROIs) and found decreased RSFC between the right MFC and right inferior parietal gyrus (IPC) and between the right IFG and left orbital prefrontal cortex (OFC) in PI patients **(A)**. The RSFC between the right MFC and right IPC negatively correlated with SRSS score. The RSFC showed negative correlation with the SRSS, ISI, SAS, and SDS scores between the right IFG and left OFC in PI patients **(B)**.

Using the right MFC and right IFG as RIOs, two-sample t-tests revealed significantly lower RSFC between the right MFC and right inferior parietal gyrus (IPC) in the PI group compared to the control group. RSFC between the right IFG and left OFC was significantly weakened in the PI group (Figure 3A, *p* < 0.05, FWE corrected).

### Correlation Analysis Results

The GM volume of the right MFC negatively correlated with SRSS score in PI patients (Figure 2A, *p* = 0.003). The GM volume of the right IFG negatively correlated with the SRSS score (Figure 2B, *p* < 0.001) and ISI score (Figure 2C, *p* = 0.006). The abnormalities in functional connectivity were associated with the scores of the sleep self-questionnaires. The RSFC between the right MFC and right IPC negatively correlated with the SRSS score (Figure 3B, *p* = 0.035). The RSFC between the right IFG and left OFC negatively correlated with the SRSS score (Figure 3B, *p* = 0.002), ISI score (Figure 3B, *p* = 0.036), SAS score (Figure 3B, *p* = 0.027) and SDS score (Figure 3B, *p* = 0.038).

## Discussion

The present study combined the VBM and RSFC methods to investigate structural and functional changes in PI patients. Compared to healthy controls, the volume of GM in several brain regions was decreased in PI patients, i.e., the left DLPFC, left OFC, bilateral MFC, right IFG, and left inferior temporal gyrus (Figure 1). The GM volume in the right MFC negatively correlated with the SRSS score (Figure 2A), and GM volume in the right IFG negatively correlated with the SRSS score (Figure 2B) and ISI score (Figure 2C). PI patients showed decreased RSFC between the right IPC and right MFC, and between the left OFC and right IFG (Figure 3A). The RSFC between the right MFC and right IPC negatively correlated with SRSS scores, and the RSFC between the right IFG and left OFC negatively correlated with ISI, SRSS, SAS, and SDS scores (Figure 3B).

Previous studies consistently confirmed the structural deficits in the prefrontal cortex (PFC) in PI subjects (Altena et al., 2010; Stoffers et al., 2012; Joo et al., 2013; Li et al., 2018). We found a decrease in the volume of GM in the DLPFC, including the middle and inferior prefrontal regions, in the PI patients. Consistent with previous findings (Joo et al., 2013; Li et al., 2018), PI patients showed a decreased GM volume in the MFC and IFG. As a key node of the default mode network (DMN), the MFC is related to the onset and maintenance of sleep (Koenigs et al., 2010). The decreased GM volume and lower regional homogeneity of MFC was associated with PI in previous studies (Joo et al., 2013; Dai et al., 2014). Recent studies revealed that the IFG is a main cortical hub in the brain network affected by PI (Yan et al., 2018). Stoffers et al. found that subjects with lower GM density in the left IFG reported more early morning awakening in an independent sample of people not diagnosed with insomnia, which may be translated into complaints of PI (Stoffers et al., 2012). Jiang et al. examined low frequency fluctuations (ALFFs) and observed lower ALFF values in the left IFG, and the PI duration negatively correlated with the ALFF value of the left IFG (Jiang et al., 2016). Recent studies observed a decrease in the DC value of the left IFG, and the DC value of PI patients positively correlated with PSQI (Yan et al., 2018). Therefore, we speculated that the IFG was a vulnerable area in the pathological process of PI. One recent study showed that sleep was susceptible to GM deficits in the PFC, and abnormality of the DLPFC may be related to PI complaints, such as early rising and difficulty falling asleep (Li et al., 2018). The PFC is associated with higher-order cognitive function, including decision-making and executive ability (Gläscher et al., 2012; McNamee et al., 2013; Squire et al., 2013). A study of a vigilance task that was associated with decision-making found that PI patients responded more slowly than the control group (Altena et al., 2008). Decision-making in previous studies was very sensitive to sleep deprivation (Vinod et al., 2007). Therefore, insomnia may be related to the abnormal volume of GM in the PFC, which leads to a decline of decision-making ability.

The SRSS of patients with PI negatively correlated with GM volumes in the MFC, and the IFG GM volumes negatively correlated with ISI (Figure 2). These preliminary results showed a significant negative correlation, which may support a relationship between the PFC and sleep quality.

Our study investigated changes in brain RSFC with structural deficits. RSFC may reveal interregional functional changes *via* the calculating of the temporal correlation between the internal fluctuations observed in spatially different brain regions in resting states (Fox and Raichle, 2007; Biswal et al., 2010). Compared with the healthy controls, PI patients showed weakened RSFC between the right MFC and right IPC and between the right IFG and left OFC (Figure 3A). The IPC is a large and heterogeneous region and an important node of the DMN. Previous sleep deprivation studies found that spontaneous activity in the IPC region and its connection patterns with other DMN subregions were abnormal after 72 h of sleep deprivation (Dai et al., 2015). These studies suggest that abnormal spontaneous activity and connectivity in the IPC are associated with sleep disorders. Horovitz et al. (2009) found that the RSFC between IPC and MFC decreased after deep sleep or partial sleep deprivation (Sämann et al., 2010). Li et al. (2014) used rs-fMRI and seed-based connectivity analysis and found that the connection between the bilateral parietal lobe and right frontal lobe was weak in PI patients. They suggested that the reduced interaction between the parietal and frontal lobes were responsible for spatial and verbal working memory deficits caused by insomnia (Walter et al., 2003). The activation of frontal and parietal lobes was lower in PI patients in a spatial memory task (Li et al., 2016). Many studies confirmed that PI patients had defects in attention processes and working memory tasks (Fortier-Brochu et al., 2012; Drummond et al., 2013). Previous research found that the frontoparietal network (FPN) was closely related to attention-keeping and working memory (Corbetta and Shulman, 2002; Seeley et al., 2007). Therefore, connection dysfunction in the FPN may be related to the pathophysiology of cognitive impairment in PI. We also observed that the significant reduction in FPN RSFC in PI patients was related to poor sleep quality, as assessed by SRSS, which may further explain the underlying neurobiological mechanism of FPN RSFC and insomnia.

We discovered that PI patients showed decreased RSFC between the right IFG and left OFC compared to good sleepers. Notably, the RSFC between the right IFG and left OFC negatively correlated with SRSS and ISI scores. The relationship between OFC and sleep quality was most frequently assessed using MRI in the sleep literature (Sexton et al., 2014). A VBM study demonstrated that PI patients had significantly reduced GM density in the left OFC (Altena et al., 2010). More importantly, the decreased GM density of the left OFC may represent a pre-existing vulnerability to sleep complaints (Stoffers et al., 2012). FMRI studies found a weakened amplitude of low-frequency fluctuations (ALFF) in the OFC after sleep deprivation (Lei et al., 2015). The OFC involves the ability to make decisions and solve problems, and it is related to sleep deprivation (Venkatraman et al., 2007). The OFC region monitors the intensity of heat stimulation in the surrounding environment (Rolls et al., 2008). Therefore, decreased activation of the OFC of PI patients may be related to their weakened understanding of optimal sleep comfort environment temperature. The decreased right IFG – left OFC RSFC correlated with SAS and SDS scores, which indicates a possible link between the RSFC of IFG-OFC with depression and anxiety. The OFC is a core brain area of the emotional arousal and motivation brain network (Olausson et al., 2007). Abnormalities in the OFC in emotion disorders were demonstrated using functional neuroimaging (Rolls, 2019). Previous research found that the lateral OFC area was associated with depression because it responded to not receiving the expected rewards, which is a typical cause of sadness and depression (Rolls, 2000, 2016, 2019). Notably, a negative correlation between the SDS and RSFC between the right IFG and left OFC was also found in healthy controls. The PFC plays an important role in emotional processing (Etkin et al., 2011). Our current study provides evidence that impaired connectivity of the PFC, especially the MFC and IFG, may be related to poor sleep quality in insomnia. However, connectivity in the IFG may be associated with depression in normal people.

However, there are some limitations in the current study. First, this cross-sectional study cannot lead to causal conclusions between PI and structural and functional abnormalities of the brain. Longitudinal studies may be performed in the future to determine whether these changes are a cause of insomnia or a consequence of long-term sleep deprivation. The number of subjects in this paper may be too small.

## Conclusion

In summary, our study used a multimodal approach that combined VBM analysis and resting-state functional connection analysis to investigate structural and functional abnormalities in the brains of PI patients. We found structural changes in the right MFC and right IFG accompanied by RSFC changes. These results indicate that VBM and RSFC effectively evaluated changes in PI-related brain structure and functional connectivity. We hope our research provides some theoretical basis for the study of PI neuropathology and ideas for clinical application.

## Data Availability Statement

The datasets generated for this study are available on request to the corresponding author.

## Ethics Statement

The studies involving human participants were reviewed and approved by the Research Ethical Committee of First Affiliated Hospital of Baotou Medical College, Inner Mongolia University of Science and Technology approved the trial. The patients/participants provided their written informed consent to participate in this study.

## Author Contributions

DX and FD: study design, data analysis, and manuscript writing. XW, CL, and HQ: data collection. TX: data analysis. KY, BL, and YH: manuscript revision. DY: study design. All authors contributed to the article and approved the submitted version.

## Conflict of Interest

The authors declare that the research was conducted in the absence of any commercial or financial relationships that could be construed as a potential conflict of interest.

## Footnotes

1 http://afni.nimh.nih.gov/

2 http://fsl.fmrib.ox.ac.uk/fsl/

3 http://www.restfmri.net/forum/REST
