# Supplementary material for: Functional Connectivity Abnormalities of Brain Regions With Structural Deficits in Primary Insomnia Patients
Source: Front Neurosci. 2020 Jun 26;14:566. doi: 10.3389/fnins.2020.00566 (PMC7332723; doi:10.3389/fnins.2020.00566)
Supplement: Supplementary file 1 [file Table_1.DOCX]

Table S1 The full results of correlation analysis

|  | PSQI | SRSS | ISI | SAS | SDS |
| --- | --- | --- | --- | --- | --- |
| MFC GM volume | r=-0.285 | r=-0.504 | r=-0.242 | r=-0.157 | r=-0.110 |
|  | p=0.108 | P=0.003 | p=0.175 | p=0.383 | p=0.543 |
| IFG GM volume | r=-0.310 | r=-0.635 | r=-0.479 | r=-0.236 | r=-0.277 |
|  | p=0.079 | P=0.001 | p=0.006 | p=0.187 | p=0.118 |
| MFC-IPC RSFC | r=-0.153 | r=-0.368 | r=-0.135 | r=-0.127 | r=-0.55 |
|  | p=0.395 | p=0.035 | p=0.455 | p=0.488 | p=0.763 |
| IFG-OFC RSFC | r=-0.093 | r=-0.519 | r=-0.366 | r=-0.384 | r=-0.362 |
|  | p=0.609 | p=0.002 | p=0.036 | p=0.027 | p=0.038 |
